# Correction: More effective drugs lead to harder selective sweeps in the evolution of drug resistance in HIV-1

**DOI:** 10.7554/eLife.24879

**Published:** 2017-01-19

**Authors:** Alison F Feder, Soo-Yon Rhee, Susan P Holmes, Robert W Shafer, Dmitri A Petrov, Pleuni S Pennings

Feder AF, Rhee S-Y, Holmes SP, Shafer RW, Petrov DA, Pennings PS. 2016. More effective drugs lead to harder selective sweeps in the evolution of drug resistance in HIV-1. *eLife* **5**:e10670. doi: 10.7554/eLife.10670.

Published 14, February 2016

We discovered an error in calling drug resistance mutations (DRMs) in the protease gene in 140 sequences in our dataset (out of 6,717 sequences). This error was upstream to nearly all analysis, so multiple figures have been updated to reflect this correction. There were also several errors in description of the analysis. In all cases, the resulting changes are minor and do not substantially change the conclusions and in some cases make them stronger.

The corrected Figure 2 is shown here:

**Figure 1:**
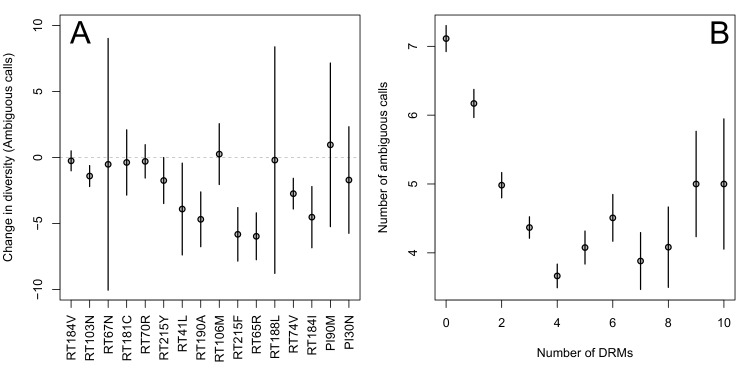

The originally published Figure 2 is also shown for reference:

**Figure 2:**
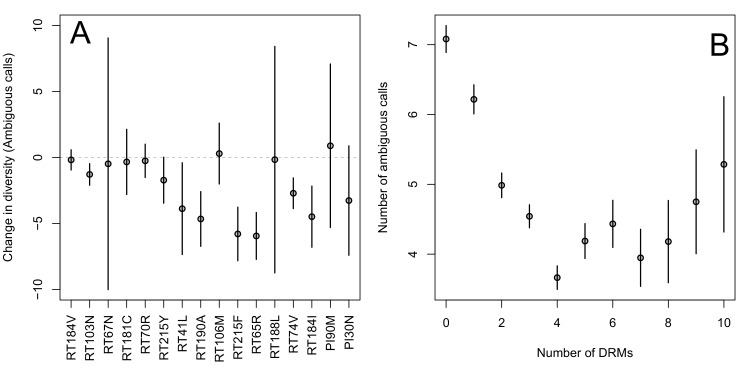

The corrected Figure 2—﻿figure supplement 1 is shown here:

**Figure 3:**
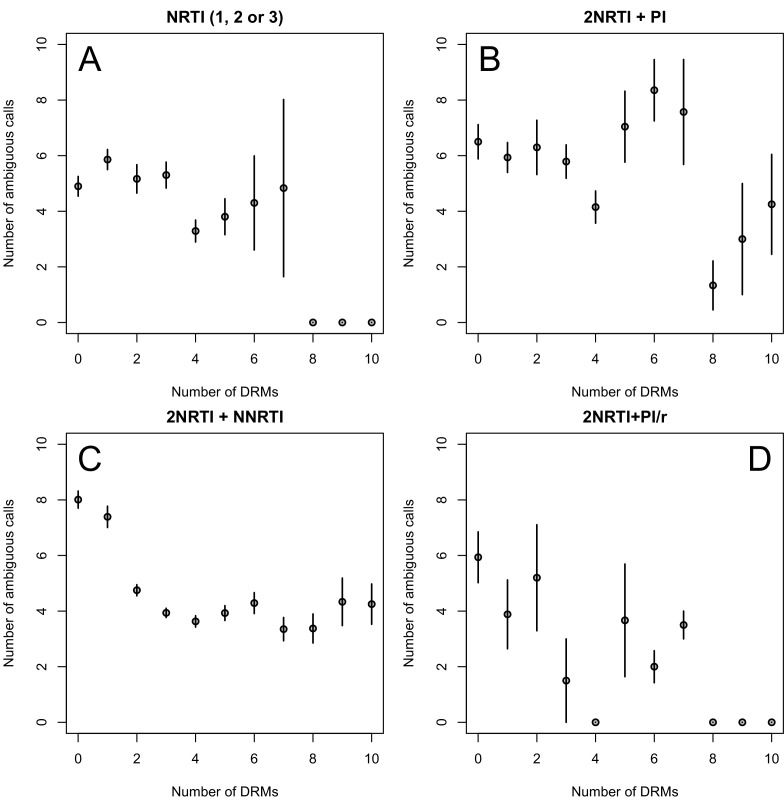

The originally published Figure 2—﻿figure supplement 1 is also shown for reference:

**Figure 4:**
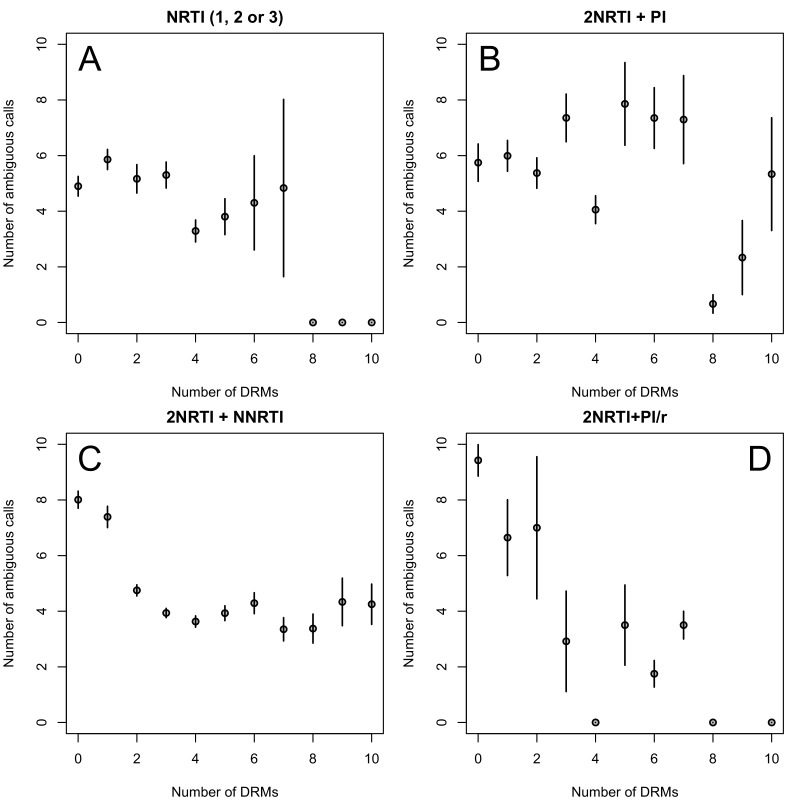

The corrected Figure 2—﻿figure supplement 2 is shown here:

**Figure 5:**
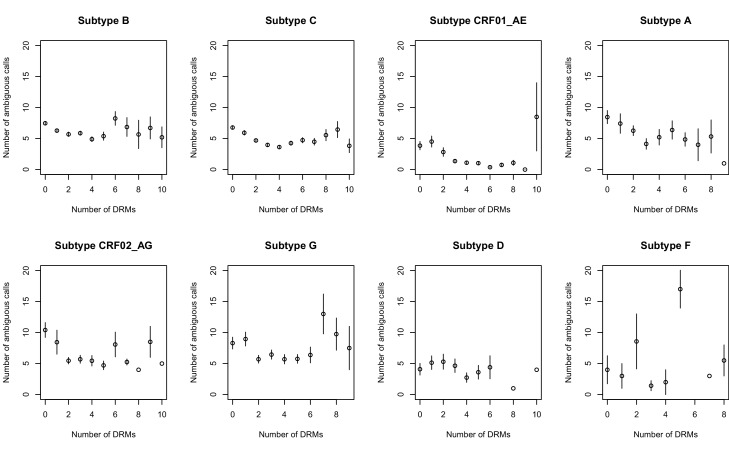

The originally published Figure 2—﻿figure supplement 2 is also shown for reference:

**Figure 6:**
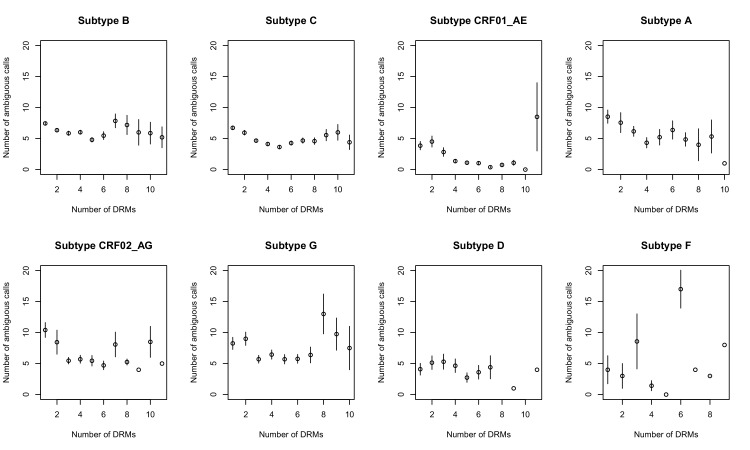

The corrected Figure 3 is shown below. Note, the two ATV/r treatments are now excluded because they do not pass the threshold requirements described in the abundant treatment dataset section of the Materials and methods.

**Figure 7:**
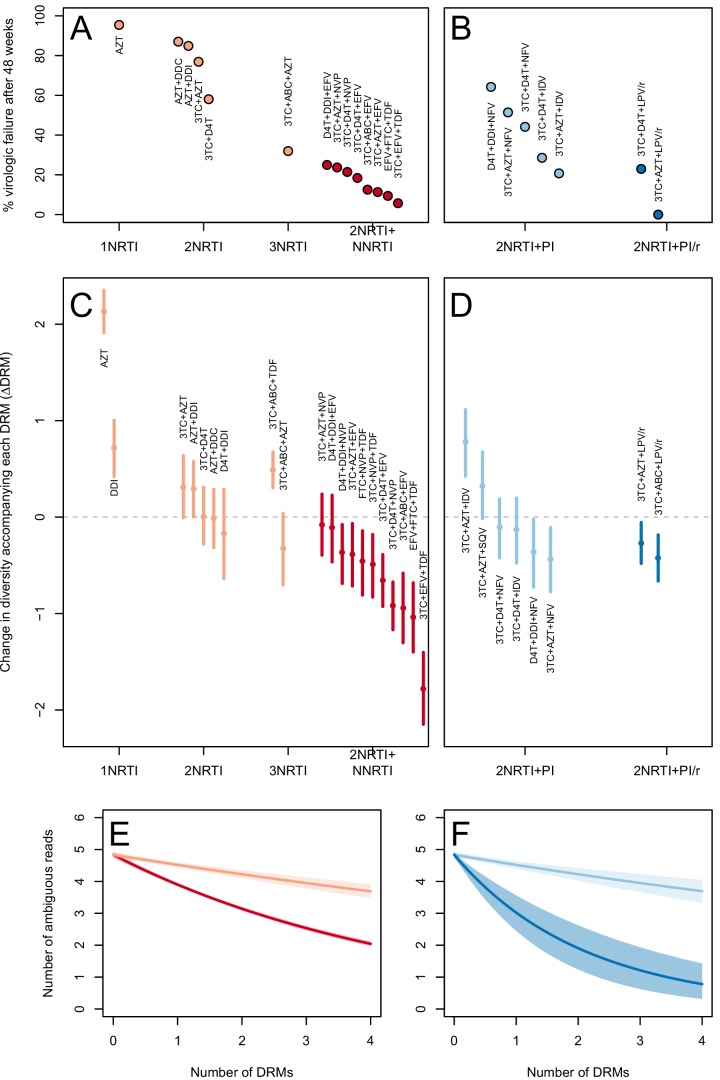

The originally published Figure 3 is also shown for reference:

**Figure 8:**
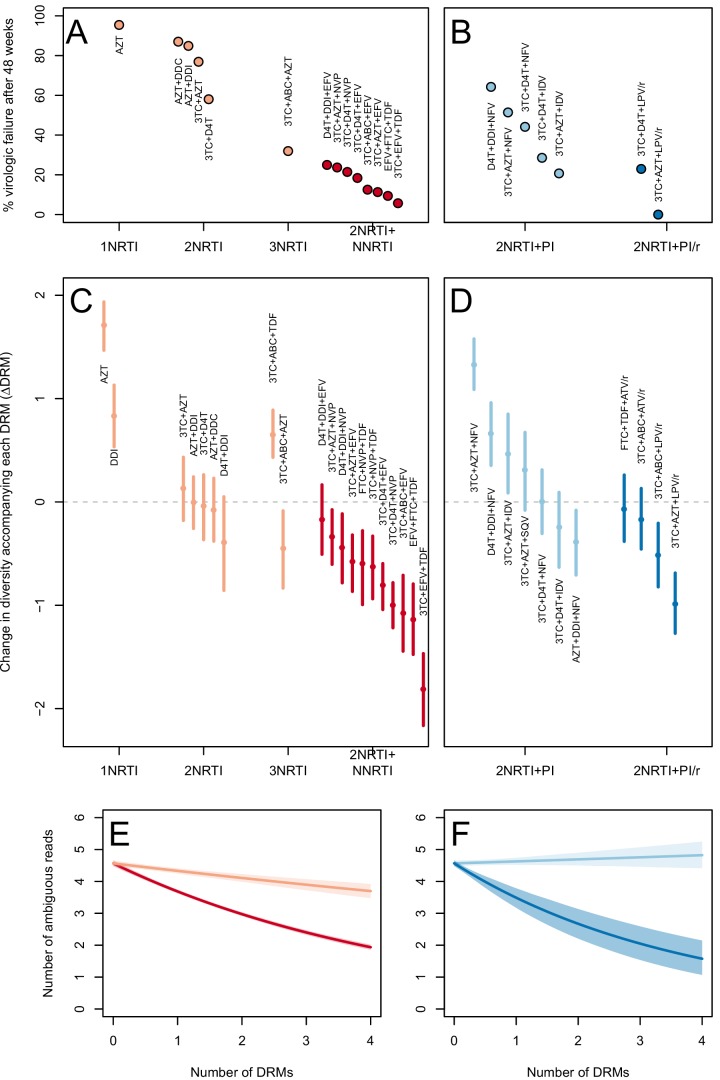

The corrected Figure 3—figure supplement 1 is shown here:

**Figure 9:**
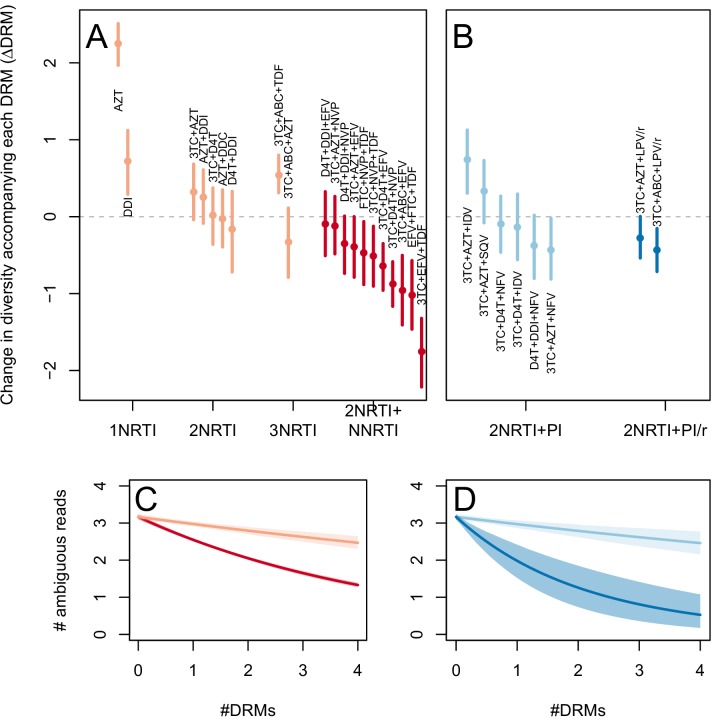

The originally published Figure 3—figure supplement 1 is also shown for reference:

**Figure 10:**
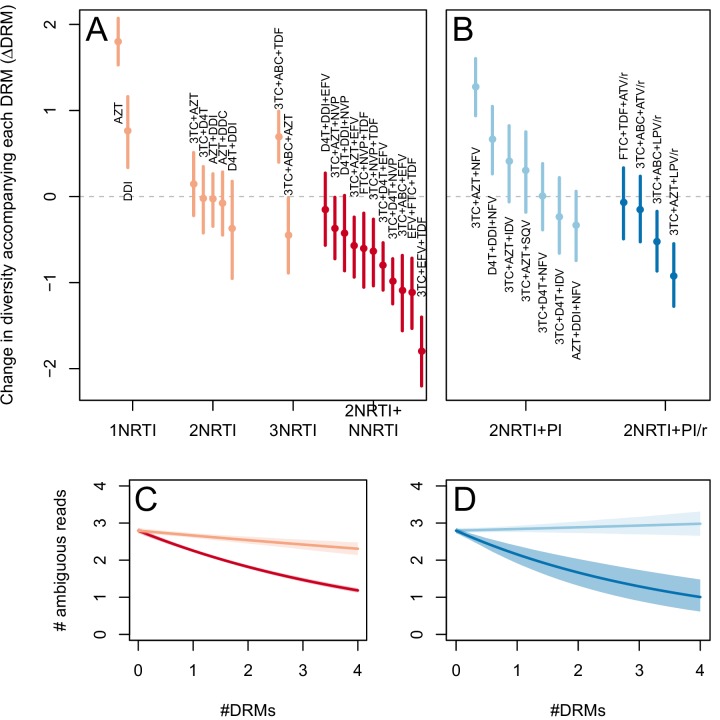

The corrected Figure 3—figure supplement 2 is shown here:

**Figure 11:**
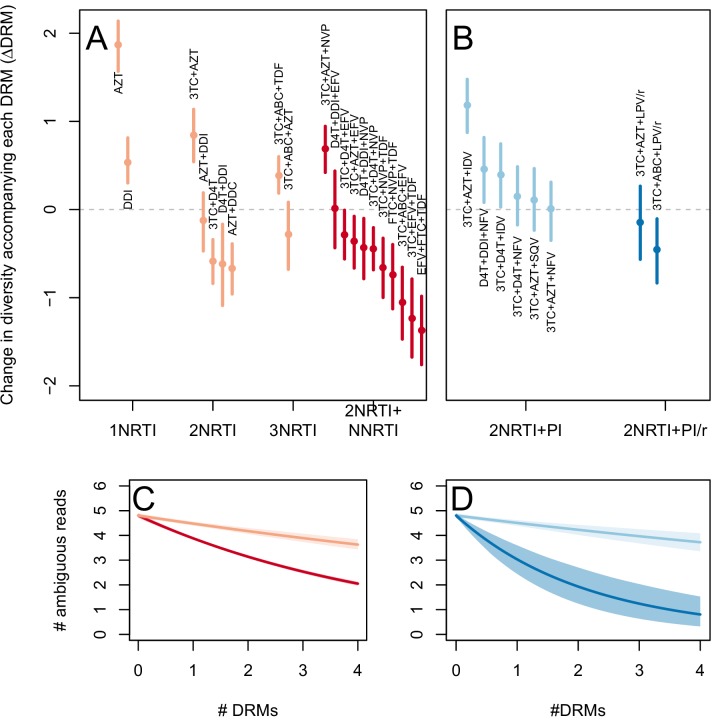

The originally published Figure 3—figure supplement 2 is also shown for reference:

**Figure 12:**
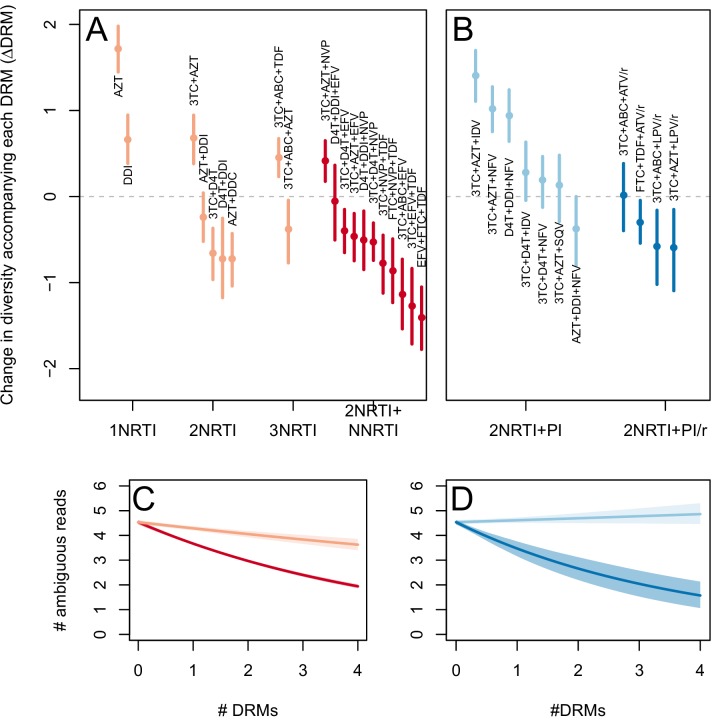

The corrected Figure 4 is shown here:

**Figure 13:**
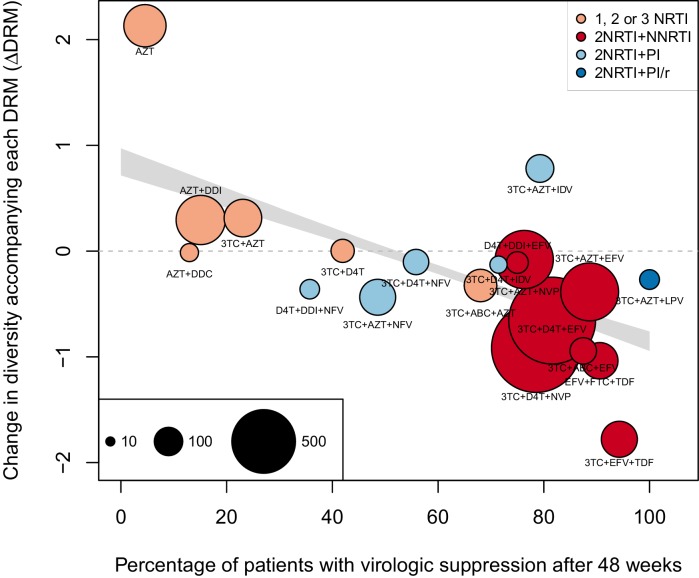

The originally published Figure 4 is also shown for reference:

**Figure 14:**
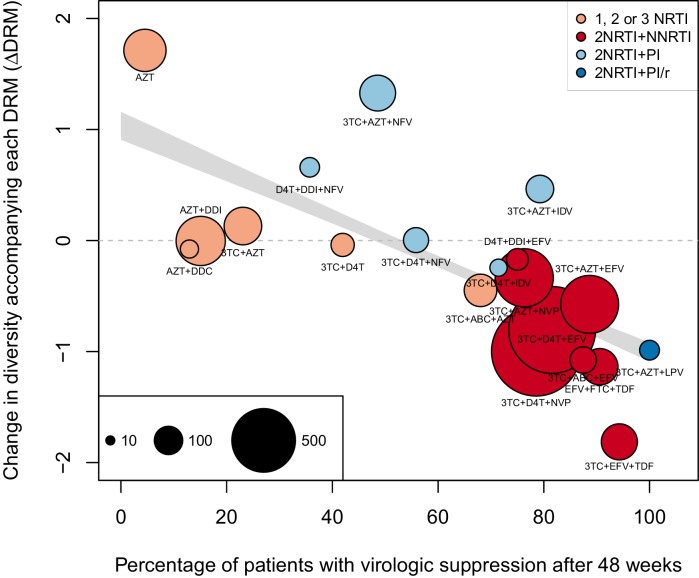

The corrected Figure 4—figure supplement 1 is shown here:

**Figure 15:**
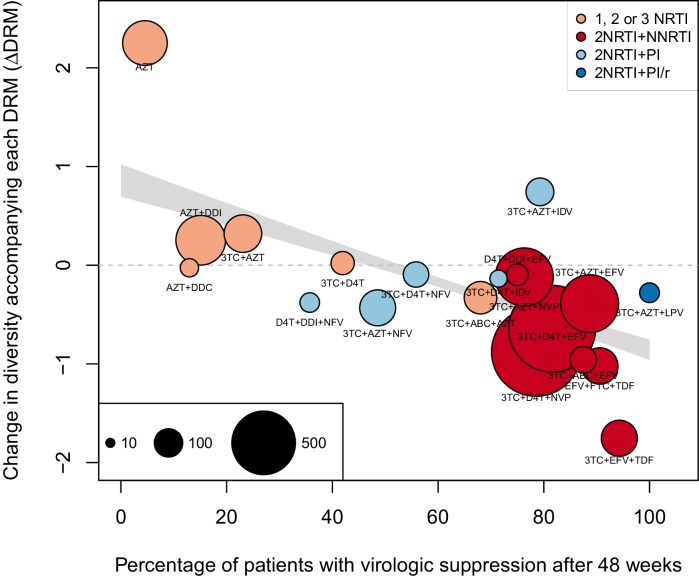

The originally published Figure 4—figure supplement 1 is also shown for reference:

**Figure 16:**
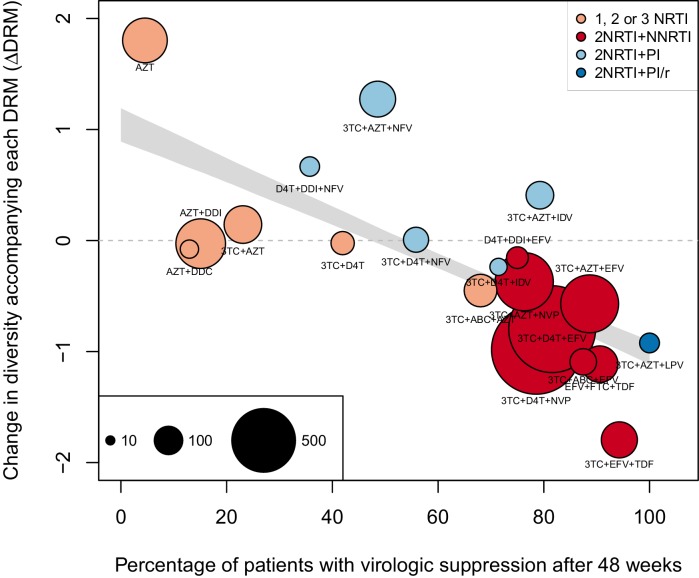

The corrected Figure 4—figure supplement 2 is shown here:

**Figure 17:**
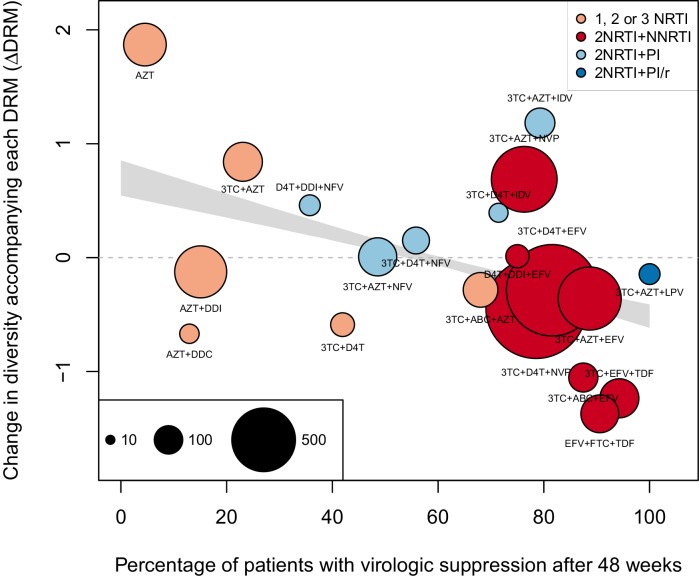

The originally published Figure 4—figure supplement 2 is also shown for reference:

**Figure 18:**
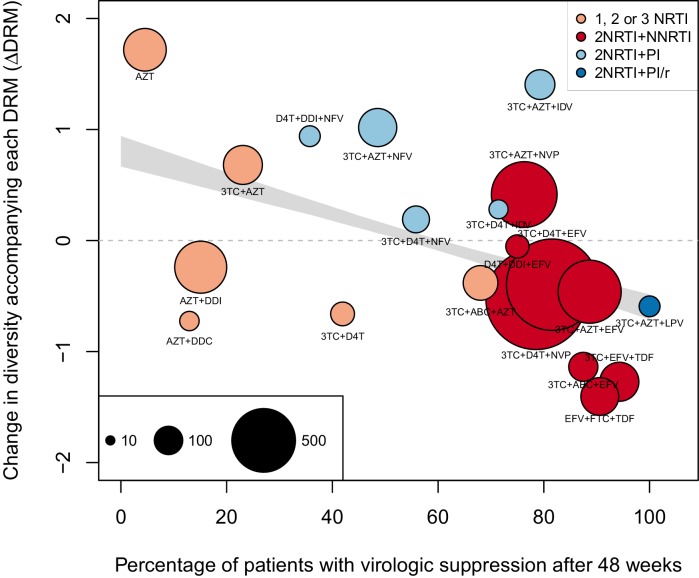

The corrected Figure 5 is shown below. Note, the two ATV/r treatments are now excluded because they do not pass the threshold requirements described in the abundant treatment dataset section of the Materials and methods.

**Figure 19:**
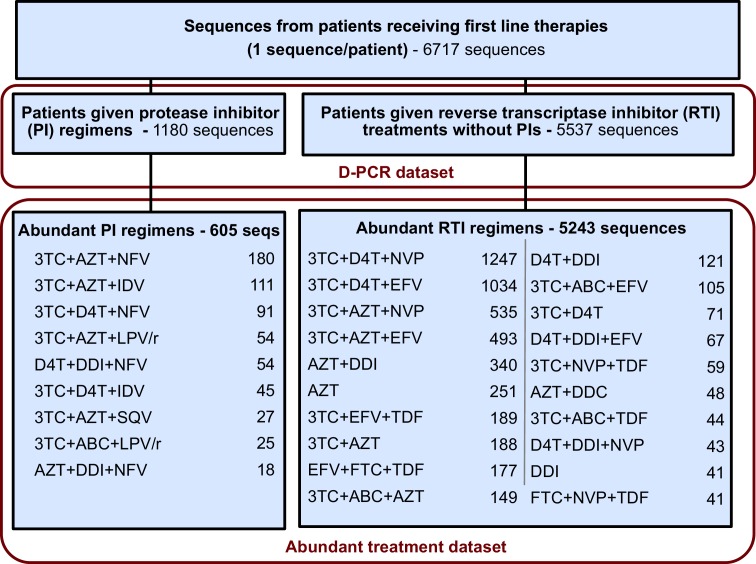

The originally published Figure 5 is also shown for reference:

**Figure 20:**
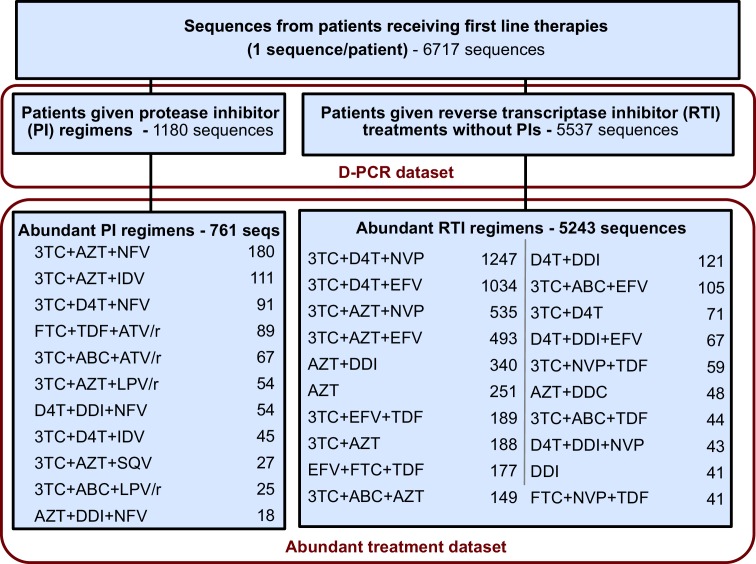

The corrected Figure 5—figure supplement 1 is shown below:

**Figure 21:**
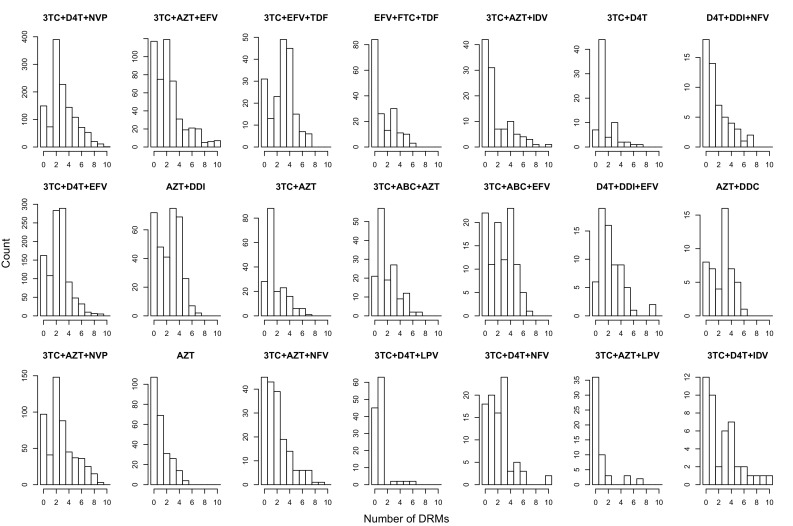

The originally published Figure 5—figure supplement 1 is also shown for reference:

**Figure 22:**
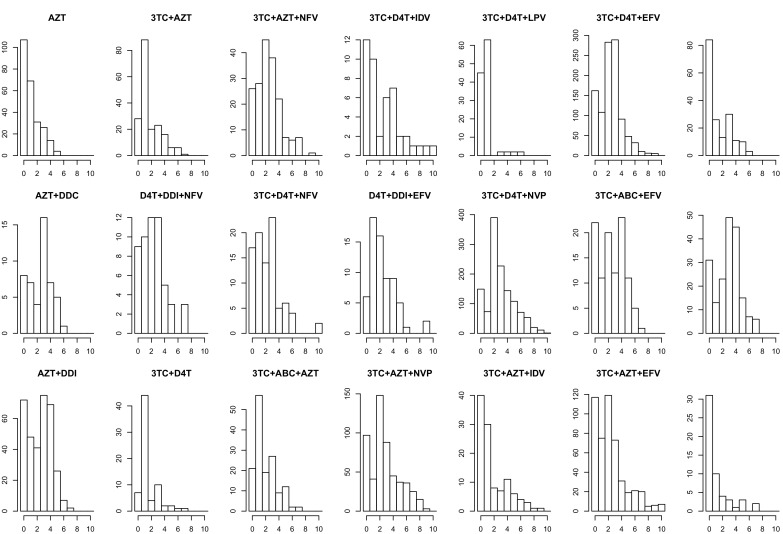

The corrected Figure 5—figure supplement 3 is shown here:

**Figure 23:**
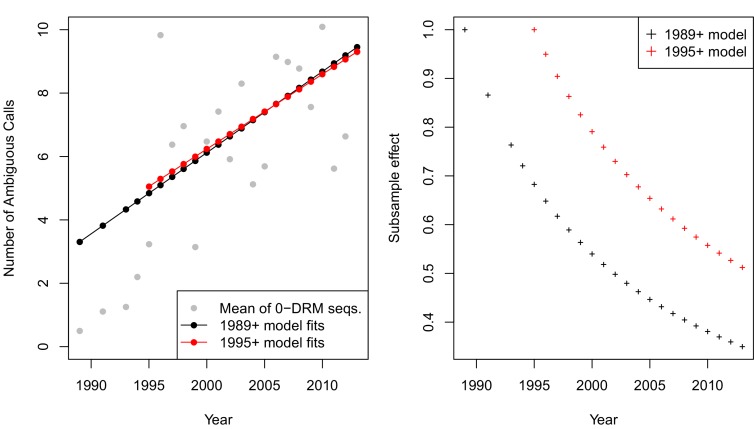

The originally published Figure 5—figure supplement 3 is also shown for reference:

**Figure 24:**
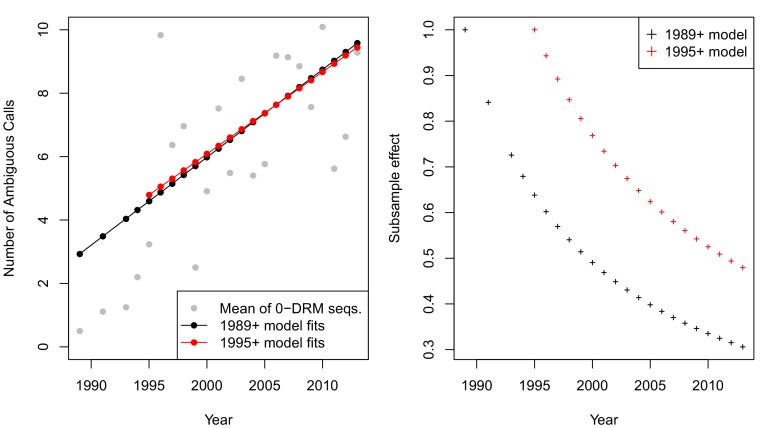

The corrected Figure 5—figure supplement 4 is shown here:

**Figure 25:**
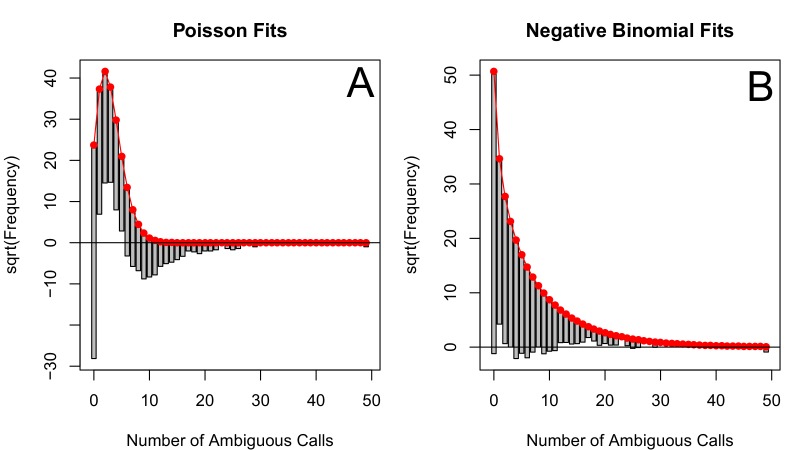

The originally published Figure 5—figure supplement 4 is also shown for reference:

**Figure 26:**
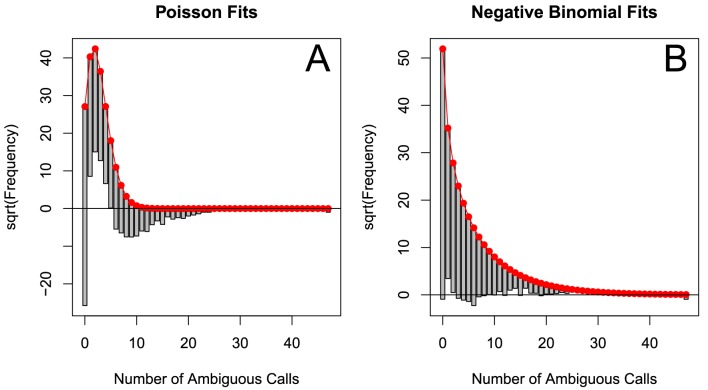

The corrected Figure 5—figure supplement 5 is shown here:

**Figure 27:**
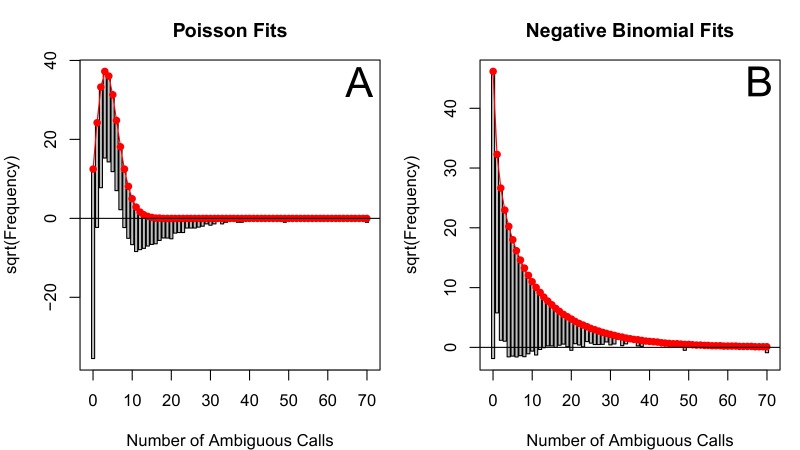

The originally published Figure 5—figure supplement 5 is also shown for reference:

**Figure 28:**
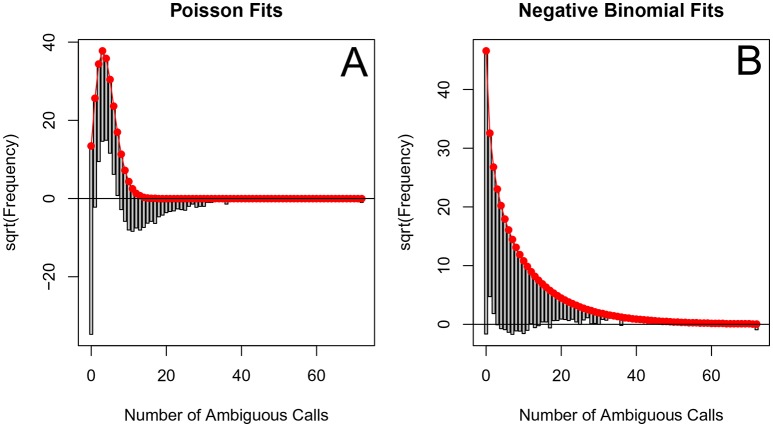

The corrected Table 1 is shown here:

**Table:** 

	αall (Intercept)	∆ (Number of DRMs)	Ɣ (Length)
1995+, ≤4 DRMS	-0.78	-0.16	0.0030
	(-0.91,-0.65)	(-0.17,-0.14)	(0.0029,0.0032)
1995+, All DRMS	-0.89	-0.097	0.0030
	(-1,-0.77)	(-0.11,-0.088)	(0.0029,0.0032)
1989+, ≤4 DRMS	-1.20	-0.15	0.0030
	(-1.4,-1.1)	(-0.17,-0.14)	(0.0028,0.0032)

The originally published Table 1 is shown here for reference:

**Table:** 

	αall (Intercept)	∆ (Number of DRMs)	Ɣ (Length)
1995+, ≤4 DRMS	-0.82	-0.12	0.0030
	(-0.94, -0.68)	(-0.13, -0.10)	(0.0028,0.0031)
1995+, All DRMS	-0.88	-0.083	0.0030
	(-1.00, -0.76)	(-0.094, -0.073)	(0.0028,0.0031)
1989+, ≤4 DRMS	-1.33	-0.12	0.0030
	(-1.50, -1.16)	(-0.13, -0.097)	(0.0028,0.0032)

The corrected Table 2 is shown here: In addition, we corrected a typo in the column headers which had ∆_1,2,3NRTI_and ∆_2NRTI+NNRTI_ switched in the original version.

**Table:** 

	αall (Intercept)	Ɣ (Length)	∆_1,2,3NRTI_	∆_2NRTI+NNRTI_	∆_2NRTI+PI_	∆_2NRTI+PI/r_
1995+, ≤4 DRMS	-0.46	0.0025	-0.068	-0.22	-0.067	-0.48
	(-0.58,-0.35)	(0.0024,0.0027)	(-0.085,-0.050)	(-0.23,-0.20)	(-0.094,-0.043)	(-0.68,-0.30)
1995+, All DRMS	-0.62	0.0026	-0.049	-0.13	-0.0035	-0.20
	(-0.72,-0.51)	(0.0025,0.0028)	(-0.061,-0.036)	(-0.14,-0.12)	(-0.019,0.011)	(-0.30,-0.12)
1989+, ≤4 DRMS	-0.91	0.0026	-0.063	-0.22	-0.065	-0.48
	(-1.00,-0.78)	(0.0024,0.0027)	(-0.084,-0.044)	(-0.23,-0.20)	(-0.098,-0.034)	(-0.76,-0.27)

The originally published Table 2 is shown here for reference:

**Table:** 

	αall (Intercept)	Ɣ (Length)	∆_1,2,3NRTI_	∆_2NRTI+NNRTI_	∆_2NRTI+PI_	∆_2NRTI+PI/r_
1995+, ≤4 DRMS	-0.51	0.0025	-0.21	-0.053	-0.27	0.013
	(-0.62,-0.40)	(0.0024,0.0027)	(-0.23,-0.20)	(-0.070,-0.036)	(-0.36,-0.18)	(-0.009,0.035)
1995+, All DRMS	-0.66	0.0026	-0.13	-0.039	-0.14	0.033
	(-0.77,-0.56)	(0.0025,0.0028)	(-0.14,-0.12)	(-0.053,-0.026)	(-0.21,-0.096)	(0.019,0.047)
1989+, ≤4 DRMS	-1.03	0.0026	-0.22	-0.047	-0.27	0.016
	(-1.18,-0.88)	(0.0024,0.0028)	(-0.23,-0.20)	(-0.069,-0.025)	(-0.39,-0.17)	(-0.013,0.043)

In addition to the above tables and figures, some of the main text also needs to be updated to reflect these changes. The changes below are listed in the order in which they appear in the main text.

For the following correction, we also note that comparisons are between x and x+1 DRMs, not between x and >x DRMs as incorrectly noted in the original text.

**CORRECTED:**

Indeed, we find that for sequences that have between 0 and 4 DRMs, additional DRMs are associated with reduced genetic diversity (Figure 2B, p-value for t-test between diversity among sequences with 0 versus 1 is 7.2 × 10^-4^, between 1 and 2 is 1.6 × 10^-5^, between 2 and 3 is 1.1 × 10^-2^, between 3 and 4 is 2.5 × 10^-3^).

**ORIGINAL:**

Indeed, we find that for sequences that have between 0 and 4 DRMs, additional DRMs are associated with reduced genetic diversity (Figure 2B, p-value for t-test between diversity among sequences with 0 versus ≥ 1 is 2.8 × 10^-4^, between 1 and ≥ 2 is 7.6 × 10^-7^, between 2 and ≥ 3 is 0.16, between 3 and ≥ 4 is 1.0 × 10^-4^).

**CORRECTED:**

In 9 of 11 NNRTI-based treatment regimens, ∆DRM is significantly below 0 (Figure 3C).

**ORIGINAL:**

In 10 of 11 NNRTI-based treatment regimens, ∆DRM is significantly below 0 (Figure 3C).

**CORRECTED:**

Among treatments containing PIs, both of the effective boosted-PI treatments had ∆DRM significantly below 0 (Figure 3D). The less effective unboosted PI treatments had ∆DRM values on average closer to 0, and two of five unboosted treatments had a ∆DRM value above 0.

**ORIGINAL:**

Among treatments containing PIs, two out of four of the effective boosted-PI treatments had ∆DRM significantly below 0 (Figure 3D). The less effective unboosted PI treatments had ∆DRM values closer to 0, and three of five unboosted treatments had a ∆DRM value significantly above 0.

For the following two corrections, in addition to fixing the problems introduced by the upstream data processing, we discovered that the reported decreases in diversity had been incorrectly computed. The values listed below fix both the problem in the upstream analysis and the computation error in the text.

**CORRECTED:**

We find that among sequences from patients receiving highly effective treatments with NNRTIs each fixed DRM is associated with a mean additional 13.6% reduction in diversity as compared to populations with no DRMs (95% confidence interval, 13.0%–14.3%). Among sequences from patients receiving less effective treatments with only NRTIs, each fixed DRM is associated with a mean additional 4.3% reduction in diversity compared to populations with no DRMs (95% confidence interval, 3.2%–5.3%) (Table 2).

**ORIGINAL:**

We find that among sequences from patients receiving highly effective treatments with NNRTIs each fixed DRM is associated with a mean additional 49.5% reduction in diversity as compared to populations with no DRMs (95% confidence interval, 49.0%–50.2%). Among sequences from patients receiving less effective treatments with only NRTIs, each fixed DRM is associated with a mean additional 15% reduction in diversity compared to populations with no DRMs (95% confidence interval, 13%–18%) (Table 2).

**CORRECTED**:

Among PI-based treatments, we found that sequences from effective treatments based on boosted PIs showed a mean additional reduction in diversity of 30.3% with each fixed DRM as compared to populations with no DRMs (95% confidence interval, 19.0%–42.3%). In contrast, sequences from less effective treatments based on unboosted PIs showed a smaller decrease in diversity of 4.3% (95% confidence interval, 2.7%-6.0%) with each fixed DRM (Table 2).

**ORIGINAL**:

Among PI-based treatments, we found that sequences from effective treatments based on boosted PIs showed a mean additional reduction in diversity of 57% with each fixed DRM as compared to populations with no DRMs (95% confidence interval, 46%–68%). In contrast, sequences from less effective treatments based on unboosted PIs showed a slight mean increase in diversity by 4% with each fixed DRM (Table 2), although this increase was not significant (95% confidence interval, -10% to 1% decrease).

**CORRECTED**:

We observe a negative relationship between ∆DRM and treatment effectiveness (Figure 4). We find that a 10% increase in treatment effectiveness is associated with 0.17 fewer ambiguous nucleotide calls with each DRM (95% confidence interval across 1000 fits, [-0.15, -0.19]). This means that patients given treatments with 50% effectiveness have approximately the same amount of diversity whether they have 0 or 3 DRMs, but patients on treatments with 80% effectiveness have 54% fewer if they have 3 DRMs as compared to 0.

**ORIGINAL**:

We observe a negative relationship between ∆DRM and treatment effectiveness (Figure 4). We find that a 10% increase in treatment effectiveness is associated with 0.2 fewer ambiguous nucleotide calls with each DRM (95% confidence interval across 1000 fits, [-0.22, -0.18]). This means that patients given treatments with 50% effectiveness have approximately the same amount of diversity whether they have 0 or 3 DRMs, but patients on treatments with 80% effectiveness have 52% fewer if they have 3 DRMs as compared to 0.

**CORRECTED:**

This restriction yielded a dataset with 5848 sequences from 29 treatments and was termed the abundant treatment dataset.

**ORIGINAL:**

This restriction yielded a dataset with 6004 sequences from 31 treatments and was termed the abundant treatment dataset.

**CORRECTED:**

This effect, taken on aggregate across all sequences was not significant (*p* = 0.09, linear regression with year predicting the number of ambiguous calls). However, when examining only sequences with 0 DRMs, a strong positive correlation emerges, with each year associated with 0.56 more ambiguous calls per sequence (*p*=4.9 × 10^−8^).

**ORIGINAL:**

This effect, taken on aggregate across all sequences was not significant (*p* = 0.16, linear regression with year predicting the number of ambiguous calls). However, when examining only sequences with 0 DRMs, a strong positive correlation emerges, with each year associated with 0.27 more ambiguous calls per sequence (*p*=5.4 × 10^−12^).

Equation 1 should read:

(Number of ambiguous reads)=f(Year)=−505.92+0.26∗Year

Equation 1 previously read:

(Number of ambiguous reads)=f(Year)=−511.25+0.26∗Year

Equation 2 should read:

$$\left(Number\;of\;ambiguous\;reads\right)=f_{1995}(Year)=-465.96+0.24*Year$$

Equation 2 previously read:

$$\left(Number\;of\;ambiguous\;reads\right)=f_{1995}(Year)=-547.88+0.28*Year$$

In addition to the above corrections, each of the following sentences should reference 29 treatments instead of 31 treatments. The new number of treatments reflects that two treatments (FTC+TDF+ATV/r and 3TC+ABC+ATV/r) now do not pass our data threshold criteria to be considered “abundant treatments” as described in the materials and methods:

- To test whether a transition from soft to hard sweeps has occurred, we look at the relationship between fixed drug resistance mutations (DRMs) and genetic diversity across 31 common anti-retroviral drug regimens.
- Because this approach relies only on widely-available Sanger sequences, we were able to compare 31 different treatments, from AZT monotherapy to treatments based on boosted PIs, sampled across more than two decades (1989–2013).
- We estimate the relationship between the number of DRMs and genetic diversity by fitting a generalized linear mixed model (GLMM) with a negative binomial error distribution for our 31 abundant treatments.
- To compare how the effect of DRMs on genetic diversity varied between two groups of treatments, we fit generalized linear models (GLMs) with a negative binomial error distribution in R (Core Team, 2013) using the package pcsl (Jackman, 2015) including all sequences belonging to the 31 treatments that passed our threshold criteria (see above).

We also note the spell-checking error that a comma was placed in the following sentence instead of a period.

This is because later years are rescaled to be comparable to 1995 observations as opposed to 1989 observations. Observations from 1995 have a greater number of ambiguous calls.

The article has been corrected accordingly.

